# Personalized Sound Therapy Combined with Low and High-Frequency Electromagnetic Stimulation for Chronic Tinnitus

**DOI:** 10.3390/jpm14090912

**Published:** 2024-08-28

**Authors:** Beatrice Francavilla, Giulia Marzocchella, Arianna Alagna, Stefania Tilotta, Elisa Di Leo, Goran Latif Omer, Stefano Di Girolamo

**Affiliations:** Department of Otorhinolaryngology, University of Rome Tor Vergata, 00133 Rome, Italyarianna.alagna@ptvonline.it (A.A.); dileo.elisa@yahoo.it (E.D.L.);

**Keywords:** tinnitus, sound therapy, electromagnetic stimulation, multimodal treatment, portable device, pitch-matching, replica tinnitus, neuromodulation

## Abstract

This study investigates a novel multimodal treatment for chronic tinnitus, a condition that significantly affects quality of life, by combining personalized sound therapy with both low- and high-frequency electromagnetic wave stimulation. Conducted at Tor Vergata University Hospital in Rome, the research involved 55 patients and employed a portable medical device for therapy delivery. Treatment effectiveness was measured through the Tinnitus Functional Index (TFI), Tinnitus Handicap Inventory (THI), Visual Analogue Scale (VAS), Hyperacusis Questionnaire (HQ), and Short Form-36 Health Survey (SF-36), encompassing initial sound therapy and subsequent multimodal treatment phases. Remarkably, 73% of participants experienced notable improvements in TFI scores, with 39% reporting a significant enhancement of 13 points or more. This improvement was mirrored in secondary outcomes like THI, VAS, and HQ scores, along with certain SF-36 domains, indicating enhanced life quality and reduced tinnitus distress. The study underscored high compliance and no adverse effects, suggesting the combined therapy’s promising potential in chronic tinnitus management. The findings advocate for further research to discern the distinct contributions of each treatment modality, positing that this innovative approach could ameliorate tinnitus symptoms and improve patient well-being, confirming its safety and efficacy.

## 1. Introduction

Tinnitus, the perception of sound in the absence of an external acoustic stimulus, presents a significant clinical challenge due to its diverse etiology, perceptual characteristics, and associated symptoms [1,2]. Chronic tinnitus patients often experience frustration, annoyance, irritability, anxiety, depression, hearing difficulties, hyperacusis, insomnia, and concentration difficulties, of all which contribute to the determination of tinnitus severity [3]. Despite advancements in neuroimaging and the understanding of tinnitus pathophysiology, effective treatments remain elusive [4,5]. However, innovative therapeutic approaches hold promise in reducing tinnitus signals and improving patient outcomes [6].

The generation of tinnitus can originate from pathological changes throughout the auditory pathway, primarily associated with cochlear lesions or auditory nerve alterations, leading to abnormal neuronal activity in central auditory pathways [7]. Tinnitus pitch often mirrors the frequency spectrum of hearing loss, highlighting the relevance of hearing impairment [8]. Tinnitus-related distress involves non-auditory brain regions, with negative reinforcement activating the limbic and autonomic nervous systems, which directs attention to the tinnitus sound [9]. Combinations of altered auditory and somatosensory inputs play significant roles [10]. Importantly, not all individuals with tinnitus experience distress uniformly, emphasizing the engagement of non-auditory brain regions in evaluating tinnitus-related signals [11,12]. The absence of negative reinforcement impedes the conscious perception of this neural activity [13].

Most therapeutic options for tinnitus have proven to be largely ineffective or, at best, selectively effective, offering only limited relief to certain patient groups [14,15]. The varied etiologies and subjective perception of tinnitus significantly complicate the development of a definitive treatment, as many current strategies often focus more on managing the associated distress and stress rather than directly targeting the tinnitus itself. Consequently, the primary aim of many interventions is to improve the patient’s quality of life, rather than eliminating the symptoms. This has resulted in a wide range of treatment modalities, each addressing different facets of tinnitus-related distress. Despite these efforts, a standardized and universally effective treatment protocol remains elusive [16].

Among the various approaches, sound therapy has emerged as one of the most prevalent methods for managing tinnitus symptoms [17,18,19,20,21]. Grounded in foundational principles of the neurophysiological model [22,23,24], sound therapy is a therapeutic approach aimed at mitigating the distress and perceptual experience of tinnitus. This method involves the strategic use of external auditory stimuli to interact with and potentially modify the individual’s perception of tinnitus, aiming to reduce the prominence of tinnitus in the patient’s auditory landscape [25,26]. This approach not only seeks to distract the attention away from the tinnitus sound, but also aims to retrain the brain’s auditory processing centers to de-emphasize the tinnitus signal, thereby lessening the psychological and emotional impact of the condition. This method encompasses several subtypes, including hearing compensation, masking, reaction to sound, and pitch match therapy [27]. Pitch match therapy, in particular, involves the delivery of sounds tailored to an individual’s perceived tinnitus pitch, potentially enhancing the efficacy of sound therapy by directly addressing the specific auditory profile of an individual’s tinnitus [20,28].

Over the past five decades, the exploration of electrical stimulation as a therapeutic avenue for tinnitus has witnessed substantial growth, with a considerable body of research contributing to our understanding of its mechanisms and potential efficacy [6,29,30,31,32]. Diverse hypotheses have been proposed, suggesting mechanisms such as increased neurotransmitter flow at synapses, enhanced blood flow in the inner ear, and the synchronization of discharges in the fibers of the auditory nerve [33,34]. As a form of neuromodulation, electrical stimulation aims to modify neural firing and abnormal neuronal activity involved in tinnitus perception [35,36,37]. The specific mechanism and target of electrical stimulation remain unclear, presenting a challenge in optimizing treatment protocols. Both invasive and non-invasive electrical stimulations have been employed along the auditory pathway. Among non-invasive forms, both low and high-frequency electromagnetic waves have been explored, with low-frequency repetitive transcranial magnetic stimulation (rTMS) gaining attention, due to its inhibitory potential in the cerebral cortex and functionally related regions displaying increased activity—often associated with cochlear hearing damage, a potential cause of tinnitus [38,39,40]. However, their use requires hospital settings, high costs and specialized equipment, and may result in side-effects. Furthermore, while they can provide short-term relief, the treatment effects are often not long-lasting [15,27]. High-frequency electromagnetic waves, though less explored, present a contrasting landscape of results, with varying effectiveness reported in different studies [41,42].

In recent times, bimodal and multimodal interventions have emerged as a prospective strategy for addressing the intricate nature of chronic tinnitus and its associated symptoms [43,44]. These interventions integrate diverse therapeutic modalities, each targeting facets of tinnitus perception and distress, with the aim of providing patients with a comprehensive, holistic, and effective management strategy from various perspectives. It is crucial to acknowledge, however, that delineating the distinct contributions of each modality within a multimodal treatment can be complex, given the intricate and diverse nature of tinnitus.

Despite the potential advantages linked with multimodal interventions, the combination of personalized sound therapy with neuromodulation strategies in a multimodal treatment approach remains an area necessitating further exploration. Sound therapy stands as a widely recognized intervention for its efficacy; meanwhile, the integration of neuromodulation strategies, particularly involving electromagnetic waves, represents an area of growing research advancements, showcasing promising developments in the field.

Within this context, our study is designed to investigate the effectiveness of an innovative multimodal treatment approach employing a device that integrates low- and high-frequency electromagnetic wave stimulation with personalized sound therapy for the management of chronic tinnitus. Through the combined application of low- and high-frequency electromagnetic signals, coupled with the inclusion of pitch-matched personalized sound therapy, we aim to explore the potential of this novel approach in effectively addressing chronic tinnitus and enhancing the overall quality of life for patients. Additionally, our study will meticulously assess the device’s efficacy, patient compliance, and safety, providing valuable insights into its therapeutic impact and usability.

## 2. Materials and Methods

### 2.1. Population of Study

This monocentric prospective study comprised 55 patients who were referred to the Otolaryngology Department of Tor Vergata University Hospital in Rome, due to chronic tinnitus. The study protocol received approval from the Internal Ethics Committee of the University Hospital Tor Vergata (with protocol number T001/2022).

Patients were enrolled using the following inclusion criteria: chronic tinnitus persisting for a minimum of 6 months, age older than 18 years, Beck Depression Inventory II (BDI-II) score ≤ 29, Mini-Mental State Examination (MMSE) score ≥ 25, and normal-to-moderate hearing loss [45,46].

Exclusion criteria included tinnitus duration of less than 6 months, presence of pulsatile, fluctuant, or poorly defined tinnitus, moderate or severe hearing loss, neurological and psychiatric disorders, BDI-II score > 29, MMSE score < 25, previous history of otological and vestibular disorders (e.g., middle or external ear tumors, Ménière syndrome, sudden hearing loss, acoustic neuroma, otosclerosis), pregnancy, and any concomitant disorder that could affect the final study results.

The study adhered to the ethical principles of the Helsinki Declaration for medical research, ensuring the protection of participant’s well-being and rights. All eligible individuals provided written consent before being enrolled in the study. To reinforce the integrity and reliability of the findings, it is essential to note that all data underwent thorough monitoring, extrapolation, and certification by an external certification entity. This additional layer of scrutiny ensures the robustness and quality of the data, underscoring the commitment to scientific rigor and ethical standards in medical research.

### 2.2. Clinical Evaluation and Self-Reported Questionnaires

All 55 participants underwent a comprehensive ENT evaluation upon enrollment. This evaluation included otoscopy, pure-tone audiometry (PTA) across a range of frequencies (0.25, 0.5, 1, 2, 3, 6, and 8 kHz) to assess hearing thresholds, speech audiometry, acoustic immittance testing, otoacoustic emissions (OAEs), and Auditory Brainstem Response (ABR), to evaluate auditory and otological health. The PTA results were used to classify the severity of hearing loss according to the World Health Organization (WHO)’s Grades of Hearing Impairment [45,46], with categories based on the average thresholds at 500, 1000, 2000, and 4000 Hz: no impairment (≤25 dB), mild impairment (26–40 dB), moderate impairment (41–60 dB), severe impairment (61–80 dB), and profound impairment including deafness (≥81 dB). Additionally, cases of hearing loss localized only at high frequencies (≥3 kHz) were specifically noted. History of noise exposure was also collected.

As part of the study protocol, patients underwent a series of self-report questionnaires, specifically designed to gather comprehensive information about tinnitus in everyday life situations and to assess the disability caused by this condition. Further details on the self-reported questionnaires utilized in the study are provided in Table 1, including a summary of their specific domains and purposes in assessing various aspects of tinnitus and associated impairments.

The primary outcome measure of the study was the Tinnitus Functional Index (TFI) [47], which quantifies tinnitus-related functional limitations and emotional distress. Secondary outcome measures included the Tinnitus Handicap Inventory (THI) [48], which assesses the impact of tinnitus on the patient’s daily life, and Khalfa’s Hyperacusis Questionnaire (HQ) [49,50], which investigates sensitivity to sound. Additionally, the study utilized the SF-36 quality of life questionnaire [51] to evaluate the participant’s overall quality of life and a visual analogue scale (VAS) [52,53] to gauge tinnitus annoyance.

During the initial visit, participants completed all the self-report questionnaires, providing baseline data for comparison throughout the study. The same measures were administered during each treatment visit, allowing for the monitoring of tinnitus variations and treatment effects. Our analysis focused not only on general improvements in questionnaire scores, but also on the proportion of patients whose score improvements met or surpassed the Minimal Clinically Important Difference (MCID) [54], a threshold of improvement established in the scientific literature for each questionnaire used.

**Table 1:** Comprehensive Overview of Self-Reported Questionnaires Employed in the Study.

Acronym	Extended Name	Description and Subscale Information	Score Interpretation
THI [48]	Tinnitus Handicap Inventory	Comprises 25 questions across three subscales: Functional, Emotional, and Catastrophic. Scoring: Yes = 4, Sometimes = 2, No = 0.	0–100 scale: 0–16 (Very mild), 18–36 (Mild), 38–56 (Moderate), 58–76 (Severe), 78–100 (Catastrophic)
TFI [47]	Tinnitus Functional Index	Contains 25 items scored on an 11-point scale, divided into eight subscales: Intrusive, Sense of Control, Cognitive, Sleep, Auditory, Relaxation, Quality of Life, Emotional.	0–100 scale: 0–17 (Not a problem), 18–31 (Small problem), 32–53 (Moderate problem), 54–72 (Big problem), 73–100 (Very big problem)
HQ [49]	Hyperacusis Questionnaire	Features 3 binary questions on auditory disorders/noise exposure and 14 items on self-rating, scored on a 4-point scale: No (0), Yes, a little (1), Yes, a lot (2), Yes, quite a lot (3).	0–42 scale: 0–27 (Not indicative of hyperacusis), 28–42 (Indicative of hyperacusis)
SF-36 [52]	Short Form Health Survey 36	Encompasses eight health domains, each scored from 0 to 100 based on weighted sum: Physical Functioning, Physical Role, Bodily Pain, General Health, Vitality, Social Functioning, Emotional Role, Mental Health.	Each scale: 0 (Maximum disability) to 100 (No disability)
VAS [53]	Visual Analogue Scale	Assesses perceived tinnitus loudness on a 0–100 scale, with participants marking intensity with pencil and paper.	0–100 scale: 0 (No tinnitus) to 100 (Worst-imaginable tinnitus)

### 2.3. Tinnitus Psychoacoustic Measures

Psychoacoustic measures, also referred to as acuphenometry, are systematic tests used to quantify diverse perceptual aspects of tinnitus, providing valuable insights into the characteristics and severity of the tinnitus perception experienced by individuals [55,56,57]. While these measures require the subjective response of a patient, they represent a methodical approach to evaluating tinnitus, offering a standardized framework to assess its impact. These tests included the following:

- Pitch Matching: this procedure involved comparing the frequency of a test sound with the patient’s tinnitus frequency until a match was achieved, aiding in identifying the specific pitch of the tinnitus sensation.
- Loudness Matching: in this step, the intensity of the previously identified frequency test sound was adjusted to match the loudness of the patient’s tinnitus, enabling the determination of tinnitus loudness.
- Minimum Masking Level (MML): MML assessed the lowest intensity level at which an external masking signal could partially or completely cover up the perception of tinnitus, revealing its audibility and maskability.
- Residual Inhibition (RI) Test: residual inhibition referred to the temporary suppression or disappearance of tinnitus following exposure to masking noise. Complete or positive residual inhibition (CRI) occurred when the tinnitus completely disappeared, while partial inhibition involved a percentage reduction in tinnitus loudness.

Psychoacoustic measures complemented self-report questionnaires and subjective assessments, guiding the subsequent personalized sound-therapy settings for effective tinnitus management.

Importantly, it is essential to note that the diverse and complex nature of tinnitus experiences involves a spectrum of pitches, such as “ringing”, “hissing”, “white noise”, “humming”, “roaring”, “engine”, or others, rather than a singular or readily identifiable pure tone. In the context of this study, patients actively participated in selecting a pitch that they subjectively deemed sufficiently matched, and at least partially masked, their unique tinnitus experience. This patient-driven approach acknowledges the nuanced and subjective nature of tinnitus, embracing the inherent variability in pitch perception.

### 2.4. Device Characteristics

The multimodal treatment was delivered using a medical device (ACUFREE, Tinnitech International srl, Rome, Italy) which simultaneously emitted sound waves, low-frequency electromagnetic waves, and high-frequency electromagnetic waves through headphones equipped with inductive and capacitive circuitry.

The inductive circuit, responsible for delivering low-frequency electromagnetic waves, was integrated into the ear cup padding of the headphones. The solenoid within the inductor produced alternating lines of force at a frequency of 30,000 cycles per second (30 KHz). For the purpose of our study, the maximum induction level across all participants was uniformly set to 3 Gauss, in order to maintain the consistency of the treatment’s intensity.

High-frequency electromagnetic waves, ranging from 4 MHz to signals up to 60 MHz with minimal harmonics, were delivered through a capacitive emitter located in front of the ear cup. The capacitive emitter produced a power density of 120/140 mW per cm^2^, leading to a total power of 1.2/1.4 Watt. This specification remained constant for all individuals in the study, ensuring a uniform exposure to high-frequency electromagnetic waves.

Individually-adjusted audio signals were delivered in the form of pure tones, matching the specific tinnitus frequencies identified via psychoacoustic measures. The pure tones delivered were selected by the patient, based on their subjective judgment of similarity to their tinnitus, aligning with the inherently subjective and diverse nature of tinnitus experiences.

### 2.5. Study Design

The study was designed to encompass six distinct phases spanning a total duration of 14 weeks, as outlined below andas detailed in Table 2.

- Screening and Baseline (V0): At the onset of the study, participants underwent a comprehensive clinical and audiological evaluation. Inclusion and exclusion criteria were assessed, and informed consent was obtained after reviewing the study’s objectives and procedures.
- Start of 2 Weeks of Sound Therapy (V1): Eligible patients received the ACUFREE medical device for a two-week period focused solely on sound therapy. The device settings were personalized based on pre-treatment psychoacoustic measures, matching each patient’s tinnitus profile. Patients were instructed to self-administer the treatment at home for 18 min, twice daily, with adherence to the treatment regimen being self-reported by the patients.
- Transition to 1st month of complete treatment (V2): After completing two weeks of sound therapy, the device settings were modified to initiate a one-month complete-treatment phase, which included the activation of low- and high-frequency electromagnetic waves through the inductive and capacitive emitter. This phase was conducted in a single-blind manner, maintaining also the sound-therapy settings.
- First month evaluation, 2-week pause (V3): Upon completing the first month of complete treatment, the device was temporarily removed, and patients were instructed to discontinue the therapy for a period of two weeks. The two-week discontinuation of therapy at V3 was implemented to accurately assess the progression of tinnitus symptoms and the treatment’s effects over time. This pause allowed us to differentiate the immediate impact of the treatment phases and to avoid potential cumulative effects of electromagnetic wave exposure, ensuring a clearer understanding of the therapy’s efficacy.
- Follow-up and start of 2nd month of complete treatment (V4): A follow-up audiological visit was conducted, and the device was returned to the patient for a second cycle of one-month complete treatment.
- Second month evaluation, 2-week pause (V5): The medical device was definitively collected, concluding the 2nd cycle of complete treatment.
- Final follow-up evaluation (V6): Two weeks after the conclusion of the second cycle of complete treatment, a follow-up visit was conducted, and control psychoacoustic measures were repeated.

Due to resource constraints and the limited number of patients we could enroll (55 participants), we prioritized a consistent study cohort over additional control conditions, such as an electrical stimulation-only group or a placebo group. This decision allowed us to focus on the comparative efficacy of sound therapy alone versus the multimodal treatment. While we acknowledge the limitations of this design, these constraints were necessary to maintain a sufficient sample size to achieve reliable preliminary results.

Compliance with the treatment regimen was rigorously assessed through a combination of methods, to ensure a comprehensive understanding of participant adherence. Notably, the therapeutic device was equipped with the capability to record and store data in daily use. This feature offered a reliable measure of compliance and accurate information about each participant’s adherence to the treatment regimen. In addition to this objective data collection, participants were also asked to self-report their adherence, providing detailed documentation of their daily usage. Simultaneously, potential adverse events were systematically addressed during structured interviews, where patients provided comprehensive information regarding any side effects or concerns related to the treatment. This multifaceted strategy allowed for the accurate monitoring of compliance and the identification and management of any issues arising from the therapy, ensuring the integrity and reliability of the study’s findings.

### 2.6. Statistical Analysis

The statistical analysis for the assessment of treatment effectiveness involved the application of both parametric and non-parametric tests. Specifically, the parametric Student’s *t*-test was utilized alongside non-parametric tests such as the Wilcoxon signed-rank test and the sign test. To determine statistical significance, a significance level of 0.05 (*p* < 0.05) was employed.

All statistical analyses were conducted using the statistical software SPSS version 28.

## 3. Results

### 3.1. Population Characteristics

Among the initial fifty-five subjects enrolled in the study, five subjects did not complete the full treatment protocol. Specifically, two participants withdrew due to therapy interruption related to a COVID-19 infection, while the remaining three cited personal reasons for discontinuation. These personal reasons primarily revolved around concomitant health problems that diverted their attention away from the management of tinnitus, leading to their withdrawal from the study. The final group of patients who successfully completed the study consisted of 50 individuals; characteristics of the study population are detailed in Table 3.

Throughout the study duration, no adverse effects directly associated with the use of the multimodal treatment device were reported by any of the patients. The treatment demonstrated excellent tolerability, with all patients exhibiting high compliance with the prescribed intervention, fostering confidence in its safety and usability.

### 3.2. Self-Reported Questionnaires

**Figure 1:**
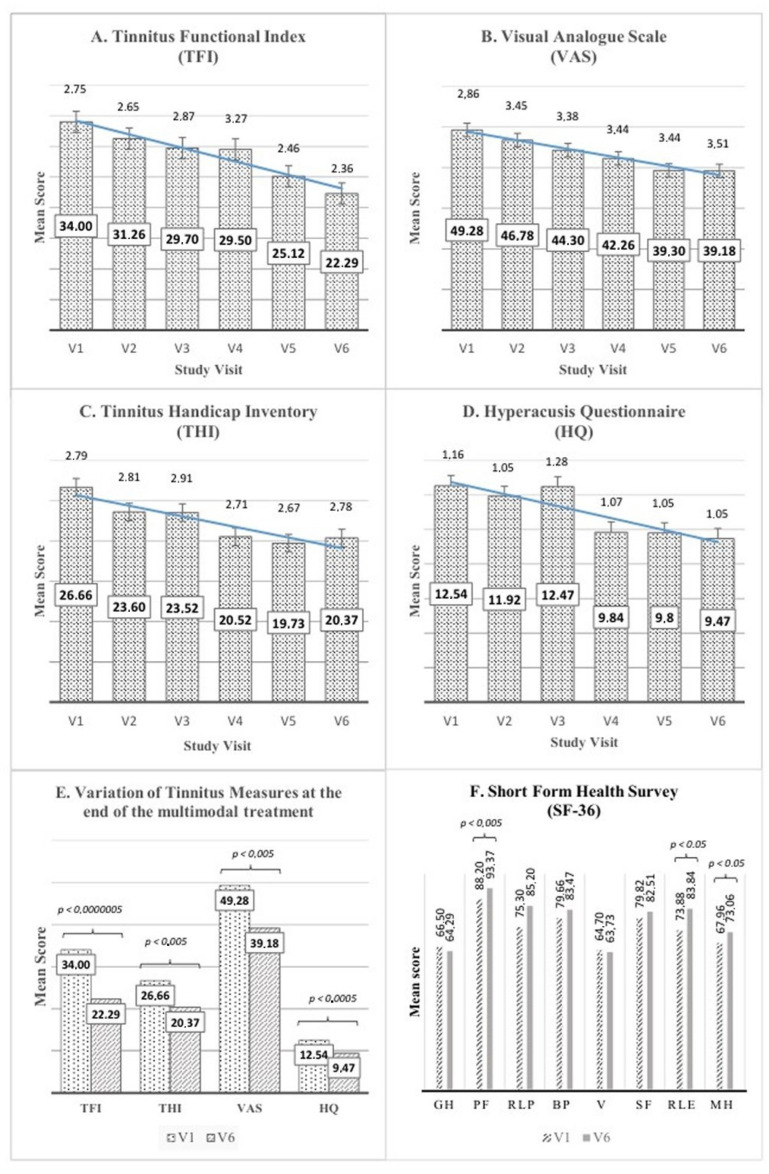
Mean Scores of Self-reported Questionnaires. (**A**) Mean scores of Tinnitus Functional Index (TFI), (**B**) Visual Analog Scale (VAS), (**C**) Tinnitus Handicap Inventory (THI), and (**D**) Hyperacusis Questionnaire (HQ) at study visits V1, V2, V3, V4, V5, and V6. The bars represent the mean values, and the error bars indicate the standard errors. Tendency lines illustrate the trends in the scores over the treatment duration. (**E**) Comparison of mean scores of TFI, THI, VAS, and HQ between study visit V1 and V6 (baseline vs. the end of treatment). The paired *t*-test revealed statistically significant reductions in tinnitus severity, related impairment, distress, and sensitivity to sound after the multimodal treatment. (**F**) Mean values of the SF-36 questionnaire subsections at baseline (V1) and after the multimodal treatment (V6), presented in individual histograms: general health perception (GH), physical functioning (PF), role limitations due to physical health problems (RLP), bodily pain (BP), vitality (VT), social functioning (SF), role limitations due to emotional problems (LRE), and mental health (MH). Paired *t*-test-derived *p*-values are shown for statistically significant differences between V1 and V6 scores. For all of the results, *p* < 0.05 was considered statistically significant.

**Figure 2:**
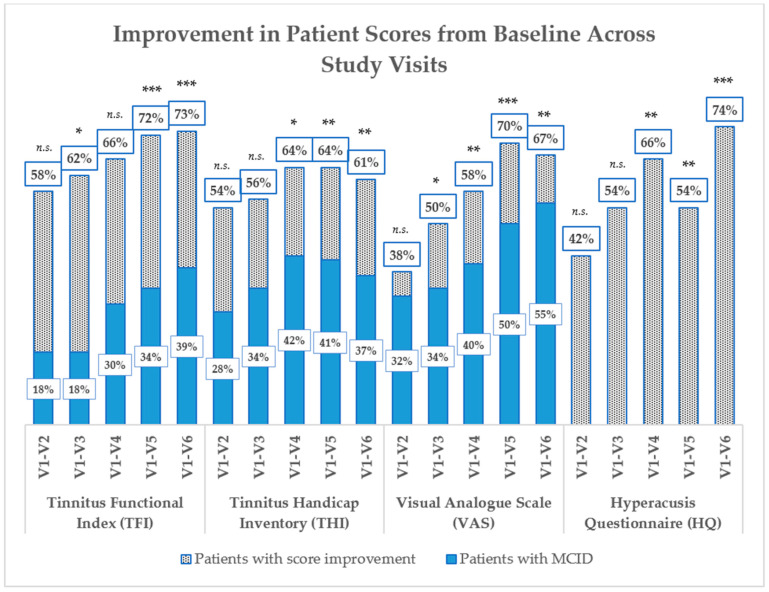
Histogram comparison of the percentage of patients exhibiting score improvements in the Tinnitus Functional Index (TFI), Tinnitus Handicap Inventory (THI), Visual Analogue Scale (VAS), and Hyperacusis Questionnaire (HQ) at sequential study visits (V1 through V6). For each questionnaire, improvements are tracked from the baseline (V1) to each subsequent visit, shown as V1–V2, V1–V3, and so forth. The black dots represent the general improvement in scores, while the blue shading indicates the subset of patients who achieved improvements that meet or exceed the Minimal Clinically Important Difference (MCID): a decrease of at least 13 points for TFI, 7 points for THI, and 10 mm for VAS. The MCID for HQ has not been established; therefore, only general improvements are depicted. Statistical significance for the final-visit comparison (V1–V6) is denoted by paired *t*-test results. The asterisks represent the statistical *p* value (* *p* < 0.05, ** *p* < 0.01, *** *p* < 0.001, n.s. indicates no significance).

This section presents the key findings from our study investigating the effectiveness of a multimodal treatment for tinnitus. Outcome measures focused on the improvement in self-reported questionnaires scores at various stages of the treatment, including a first phase (V2) with sound therapy only, an intermediate visit after one month of treatment, followed by a 2-week pause (V3 and V4, respectively), the conclusion of the multimodal treatment (V5), and the subsequent evaluation after a 2-week pause for the final follow-up (V6). Results are detailed in Table 4, Figure 1 and Figure 2.

The primary outcome measure, TFI score improvement, was assessed at different visits. At the end of the first two weeks of treatment (V2), which consisted of sound therapy only, there was no statistically significant improvement in TFI scores (*p* > 0.05), suggesting that sound therapy alone did not lead to substantial changes in tinnitus severity. However, at the conclusion of the two cycles of multimodal treatment (V6), 73% of patients exhibited a substantial reduction in TFI scores, indicating a positive and statistically significant response to the intervention (*p* < 0.01). Among the patients who responded positively to the treatment, the mean difference between the initial (V1) and final (V6) TFI scores was 17.14 points, representing a substantial reduction in tinnitus severity. Notably, 39% of patients achieved an improvement of more or equal to 13 points, a threshold considered the minimal clinically important difference (MCID), according to Meikle et al. [47,54]. At the intermediate visits (V3), which took place after one month of treatment, there was a minor improvement in TFI scores (4.30 point), with a mean score of 29.70.

In addition to the notable reduction in TFI scores at the conclusion of the treatment, significant improvements were also observed across all secondary outcome measures, including Visual Analog Scale (VAS), Tinnitus Handicap Inventory (THI), Short Form-36 Health Survey (SF-36), and Hyperacusis Questionnaire (HQ).

Visual Analog Scale (VAS) scores exhibited a noteworthy improvement, with a mean VAS change of 10.3 in the entire study group (mean VAS V1 = 49.28, mean VAS V6 = 39.18, *p* < 0.01). This signifies a substantial decrease in tinnitus-related distress, supporting the efficacy of the treatment in addressing subjective discomfort. Furthermore, at V6, 67% of patients experienced a VAS improvement, and 55% demonstrated an improvement of more than 10 mm, a threshold considered the MCID by Adamchic et al. [54,58].

Tinnitus Handicap Inventory (THI) scores displayed significant improvement, with an overall positive response observed in 61% of patients, reflecting a reduced perception of tinnitus-related impairment (*p* < 0.01). At V6, the overall study population exhibited a mean score change of 5.8, while the subgroup with THI score improvement showed a substantial mean score change of 12.8. Importantly, 37% of the study population achieved a THI improvement of 7 points or more, considered to be the MCID, according to Zeman et al. [50,54,55,56,58,59].

Hyperacusis Questionnaire (HQ) scores indicated a reduction in 74% of the study population (*p* < 0.01), with an improvement in mean score change of 3.2 points and with an improvement of more or equal to 30% in 35% of patients. While the definition of a minimal clinically relevant-score improvement for HQ is not established in the literature, and there are concerns about its consistent use as an outcome tool in tinnitus patients, this observation suggests a positive trend toward reducing sound sensitivity and enhancing tolerance to everyday sounds. Importantly, this trend was consistent with similar patterns observed in other outcome measures.

Furthermore, the treatment led to enhancements in specific domains of the Short Form-36 Health Survey (SF-36) scores, designed to assess various aspects of health-related quality of life. Specifically, the observed improvements were statistically significant in the physical functioning domain (PF, *p* < 0.01), in the role limitations due to emotional health (RLE), and in the mental health (MH) domains (*p* < 0.05). However, no significant improvements were found in other SF-36 subsections (Role limitations–physical; Bodily pain; General health; Vitality; and Social functioning). The diverse and multifaceted impact of chronic tinnitus on individuals’ lives may contribute to variations in treatment outcomes across different domains. Despite these variances, the end-of-treatment positive effects were particularly pronounced in crucial aspects of health-related quality of life. This underscores the potential of the multimodal treatment in addressing the complex challenges posed by chronic tinnitus, especially in domains related to mental well-being.

## 4. Discussion

Tinnitus remains a complex and challenging symptom to manage, and the search for effective treatments has long been ongoing [60]. In this study, we evaluated the efficacy of a novel multimodal treatment device that combines personalized sound therapy, pitch-matched to replicate the patient’s tinnitus, with low- and high-frequency electromagnetic-wave stimulation emitted through an inductive and capacitive method. In light of the growing scientific interest in multimodal therapeutic approaches for severe or complex diseases and symptoms, our findings offer valuable insights into the potential of this multimodal approach in addressing tinnitus-related distress.

In the multimodal approach employed in our study, personalized sound therapy assumed a crucial role, drawing upon the well-established efficacy of sound therapy in managing tinnitus-related distress [61,62]. In a departure from conventional methods, we introduced a technique resembling “replica tinnitus”-sound therapy—a subtype of pitch-matched sound therapy that has received limited exploration in the literature. This approach involved delivering a pure tone through the device, closely resembling the patient’s perceived pitch and guided by psychoacoustic measures. Differently from that in standardized approaches, patients actively participated in selecting a pure tone that subjectively mirrored their tinnitus. This patient-centric selection process added a level of personalization to the treatment, potentially enhancing its effectiveness. By delivering a sound that replicates the patient’s unique tinnitus perception, our approach aimed to desensitize the auditory system to the tinnitus percept. The ultimate goal was a reduction in the perception of tinnitus loudness and distress, in line with the principles of the neurophysiological model [22,23,63].

The use of low- and high-frequency electromagnetic waves as neuromodulation strategies represents an innovative approach in tinnitus management. These techniques aim to modulate the activity of neural circuits involved in tinnitus perception and processing [64]. Low-frequency stimulation may have inhibitory effects on hyperactive neurons in the auditory system, offering a potential mechanism to suppress tinnitus signals. On the other hand, high-frequency stimulation is hypothesized to exert anti-inflammatory and anti-nociceptive effects, contributing to the potential alleviation of tinnitus symptoms. The integration of these neuromodulation strategies in this multimodal treatment highlights the potential of targeting underlying neurophysiological mechanisms to mitigate tinnitus symptoms effectively.

The primary outcome measure, Tinnitus Functional Index (TFI) score, exhibited a significant reduction at the treatment’s conclusion, indicating a positive response to the intervention. The treatment’s effects were not immediately evident, as seen in the gradual response observed at the intermediate visits, emphasizing the importance of continued adherence for optimal outcomes. Secondary outcome measures also showed significant improvements. Visual Analog Scale (VAS) scores demonstrated reduced tinnitus-related distress, while Tinnitus Handicap Inventory (THI) scores improved, reflecting diminished tinnitus-related impairment. Hyperacusis Questionnaire (HQ) scores significantly improved, indicating reduced sound sensitivity and better tolerance. Regarding the Short Form-36 Health Survey (SF-36), significant improvements in role limitations due to emotional health (RLE), physical functioning (PF), and mental health (MH) were observed. However, other SF-36 domains did not show significant improvements, possibly due to individual variations in coping mechanisms, pre-existing mental health conditions, and tinnitus severity.

Our results provide preliminary evidence supporting the efficacy of a multimodal treatment approach for chronic tinnitus, integrating personalized sound therapy with low- and high-frequency electromagnetic wave stimulation. While the results indicate potential combined effects in alleviating tinnitus symptoms, it is advisable in the future to address the study’s limitations and provide a nuanced interpretation of the findings.

One limitation is the absence of an external control group, especially one incorporating sham stimulation, which would have allowed for a more rigorous evaluation of the treatment’s true effects. The lack of differentiation between the effects of personalized sound therapy and electromagnetic-wave stimulation limits our capacity to independently attribute the substantial improvements observed to each specific component. This limitation arises due to the continuous application of both modalities throughout the treatment phases, making it challenging to isolate their individual contributions. It is essential to note that the study included an internal control group (V2) exposed to sound therapy alone for the initial two weeks of the treatment. While this internal control group exhibited fewer relevant results compared to the multimodal treatment, the limited duration of sound therapy alone during this phase leaves open the possibility of a time effect contributing to the observed outcomes. Furthermore, it is important to acknowledge that in other studies, more than two weeks are often required for sound therapy to demonstrate significant effects, suggesting that the duration for V2 may have been too short to fully assess the potential benefits of sound therapy alone. Future research with extended control phases and larger sample sizes could provide a more comprehensive understanding of the individual contributions of each treatment component and the potential impact of time effects

An additional limitation of our study is the lack of neurophysiological assessments, such as the Wave V/I ABR ratio, which Schaette and McAlpine (2011) [65] identified as a marker of ‘hidden hearing loss’ linked to tinnitus. We also did not include EEG measurements to monitor neuroplastic changes [66,67,68,69,70,71]. These assessments could have provided deeper insights into the neural mechanisms and effects of our multimodal treatment. Furthermore, the study did not perform an accurate assessment of noise-exposure history, which could be a critical factor in understanding the etiology and progression of tinnitus in our subjects. Future studies should incorporate these measurements and assessments, to better understand the treatment’s impact on brain function, tinnitus management, and the role of noise exposure.

Despite the inherent challenge in isolating individual treatment effects, our study demonstrates the overall efficacy of this multimodal treatment in enhancing Tinnitus Functional Index (TFI) scores and secondary outcome measures. Moreover, our study rigorously evaluated the tolerability and safety of the tested approach, revealing no reported adverse effects throughout the study duration. The good level of patient compliance and the absence of side effects contribute to a positive assessment of the device’s usability and safety profile. An additional strength lies in the portability and home-based nature of this therapy, allowing patients to conveniently use it in the comfort of their homes. This feature may enhance treatment compliance and foster patient engagement, both critical factors for the successful management of tinnitus.

In conclusion, while acknowledging the limitations in the study design, our findings provide promising evidence for the potential of multimodal treatments in tinnitus management. The integrated approach, despite challenges in differentiating specific effects, demonstrated positive outcomes in alleviating overall tinnitus symptoms and enhancing the patient’s quality of daily life. The evaluation of the tested-treatment tolerability and efficacy, coupled with its portable and home-based attributes, strengthens its potential as a convenient and safe intervention for chronic tinnitus. Future research should aim to incorporate larger sample sizes, well-controlled designs, and differentiation strategies, to further elucidate the distinct contributions of each treatment component.

## 5. Conclusions

The integration of personalized sound therapy with electromagnetic-wave stimulation presents a promising multimodal approach to managing chronic tinnitus. Future studies should aim to isolate and evaluate the specific contributions of each treatment component. The findings suggest that this innovative approach can significantly improve tinnitus symptoms and patient quality of life, with high safety and compliance.

## Figures and Tables

**Table 2:** Detailed Schedule of the Study’s Phases and Evaluative Measures.

Visit Code and Title		Time	Evaluations and Activities
V0	Screening and baseline assessment	Day 0	ENT evaluation and anamnesis Evaluation of inclusion and exclusion criteria Pure-tone audiometry Speech audiometry Acoustic immittance test Otoacoustic emissions Auditory Brainstem Response BDI-II MMSE Enrollment of the patient Revision and signature of informed consent
V1	Start of 2 weeks of Sound Therapy	Day 0	Psychoacoustic measures Collection of self-reported questionnaires Installment of the *ACUFREE* medical device Commencement of 2 weeks of only sound therapy
V2	Transition to 1st month of complete treatment	Day 15 ± 3	Collection of self-reported questionnaires Modification of the device settings: activation of low- and high-frequency electromagnetic waves, in addition to sound therapy
V3	First month evaluation; 2-week pause	Day 45 ± 3	Collection of self-reported questionnaires Two-week pause
V4	Follow-up and start of 2nd month of complete treatment	Day 60 ± 3	Collection of self-reported questionnaires On the basis of the overall judgment of the treatment, a complete second cycle of 1 month was performed.
V5	Second month evaluation; 2-week pause	Day 90 ± 3	Collection of self-reported questionnaires Start of 2-week pause
V6	Final follow-up evaluation	Day 105 ± 3	Collection of self-reported questionnaires Follow-up psychoacoustic measures

**Table 3:** Clinical characteristics of the study population.

Characteristics	Number of Participants	Percentage
Total participants who completed the treatment	50	-
Gender		
Male	32	64%
Female	18	36%
Tinnitus Laterality		
Unilateral tinnitus	18	36%
Bilateral tinnitus	32	64%
Hearing Status		
Normal hearing	8	16%
High-frequency hearing loss	12	24%
Mild bilateral hearing loss	26	52%
Mild asymmetrical hearing loss	1	2%
Moderate bilateral hearing loss	1	2%
Moderate asymmetrical hearing loss	2	4%

**Table 4:** Changes in Self-Reported Questionnaire Scores Throughout the Study: detailed analysis of changes in scores from baseline for the Tinnitus Functional Index (TFI), Tinnitus Handicap Inventory (THI), Visual Analogue Scale (VAS), and Hyperacusis Questionnaire (HQ) across six study visits (V1 to V6). (A) Average scores, standard errors (SE), and ranges for each questionnaire at every visit. (B) Score variations between visits, expressed as mean changes ± SE, with statistical significance evaluated through paired *t*-tests. This section highlights the proportion of patients showing score improvements, with additional focus on those achieving the Minimal Clinically Important Difference (MCID) in tinnitus outcome measures.

**(A) Score Overview by Study Visit**							
		**V1**	**V2**	**V3**	**V4**	**V5**	**V6**
TFI	Mean	34.00	31.26	29.70	29.50	25.12	22.29
	SE	2.74	2.64	2.87	3.27	2.45	2.36
	Range	5–87	3–88	1–82	4–100	1–87	0–75
THI	Mean	26.66	23.60	23.52	20.52	19.73	20.37
	SE	2.79	2.81	2.91	2.71	2.67	2.77
	Range	0–86	0–78	0–94	0–94	0–96	0–92
VAS	Mean	49.28	46.78	44.30	42.26	39.30	39.18
	SE	2.86	3.44	3.38	3.43	3.44	3.51
	Range	10–81	0–100	0–100	0–100	0–98	0–98
HQ	Mean	12.54	11.92	12.47	9.84	9.80	9.47
	SE	1.16	1.04	1.27	1.06	1.04	1.05
	Range	0–30	0–26	0–36	0–26	0–23	0–23
**(B) Variations in Self-Reported Questionnaire Scores**							
		**V1–V2**	**V1–V3**	**V1–V4**	**V1–V5**	**V1–V6**	
TFI	Mean Score Change	2.74 ± 21.14	4.30 ± 1.77	4.50 ± 3.05	8.88 ± 2.30	11.36 ± 1.99	
	*p*-value	0.21	0.02	0.15	<0.01	<0.01	
	% of Patients with Improved Scores	58%	62%	66%	72%	73%	
	Mean Score Change in Improved Patients	11.24 ± 21.19	11.45 ± 11.72	14.15 ± 21.12	15.75 ± 21.23	17.14 ± 11.91	
	% of Patients with MCID (≥13-Point [44])	18%	18%	30%	34%	39%	
THI	Mean Score Change	3.06 ± 1.92	3.14 ± 1.77	6.14 ± 2.20	6.93 ± 2.20	5.57 ± 1.73	
	*p*-value	0.12	0.08	0.01	0.01	<0.01	
	% of Patients with Improved Scores	54%	56%	64%	64%	61%	
	Mean Score Change in Improved Patients	10.96 ± 21.14	10.79 ± 11.59	13.69 ± 21.34	14.47 ± 21.39	12.83 ± 11.67	
	% of Patients with MCID (≥7-Point [55])	28%	34%	42%	41%	37%	
VAS	Mean Score Change	2.50 ± 11.68	4.98 ± 21.42	7.02 ± 21.99	9.98 ± 21.77	10.28 ± 31.06	
	*p*-value	0.15	0.05	0.01	<0.01	<0.01	
	% of Patients with Improved Scores	38%	50%	58%	70%	67%	
	Mean Score Change in Improved Patients	14.11 ± 11.94	17.52 ± 21.60	17.07 ± 21.64	18.89 ± 21.55	20.27 ± 21.90	
	% of Patients with MCID (≥10-Point [54])	32%	34%	40%	50%	55%	
HQ	Mean Score Change	0.62 ± 0.71	0.32 ± 0.87	2.70 ± 0.82	2.74 ± 0.90	3.18 ± 0.81	
	*p*-value	0.39	0.40	< 0.01	< 0.01	< 0.01	
	% of Patients with Improved Scores	42%	54%	66%	54%	74%	
	Mean Score Change in Improved Patients	4.95 ± 01.75	4.48 ± 01.63	5.76 ± 01.74	7.07 ± 01.96	5.46 ± 01.74	

## Data Availability

All data will be available under request.
